# Real-time magnetic resonance-guided radiofrequency ablation and lesion evaluation in an magnetic resonance-compatible isolated beating pig heart platform

**DOI:** 10.1016/j.hroo.2025.09.014

**Published:** 2025-09-19

**Authors:** Luuk H.G.A. Hopman, Eric M. Schrauben, Jules L. Nelissen, Pieter J. Reitzema, Machteld J. Boonstra, Bart L.M. Smeets, Renate W. Boekhoven, Daniel Sunnarborg, Jouke Smink, Hans W.M. Niessen, Cornelis P. Allaart, Aart J. Nederveen, Marco J.W. Götte

**Affiliations:** 1 Department of Cardiology, Amsterdam UMC, Amsterdam, The Netherlands; 2 Department of Radiology and Nuclear Medicine, Amsterdam UMC, Amsterdam, The Netherlands; 3 LifeTec Group B.V., Eindhoven, The Netherlands; 4 Imricor Medical Systems, Burnsville, Minnesota; 5 Philips Healthcare, Best, The Netherlands; 6 Department of Pathology, Amsterdam UMC, Amsterdam, The Netherlands; 7 Division of Cardiology, Department of Cardiac Sciences, Cumming School of Medicine, Libin Cardiovascular Institute, University of Calgary, Calgary, Canada

**Keywords:** Interventional cardiovascular magnetic resonance imaging, Ablation, Histology, Real-time guidance, Pig heart, Ablation lesion

## Abstract

**Background:**

Interventional cardiovascular magnetic resonance imaging (MRI) offers real-time, radiation-free guidance for complex procedures such as myocardial ablation, marking a promising advance in electrophysiology. However, further development is limited by challenges in magnetic resonance (MR)-compatible instrument testing, MRI sequence validation, and accurate correlation with histopathology, hindered by the limitations of in vivo tissue evaluation.

**Objective:**

This study investigated the feasibility of real-time MR-guided radiofrequency (RF) ablation in an MR-compatible isolated beating pig heart platform and characterized ablation lesions using MRI and histopathology.

**Methods:**

A heart from a pig slaughtered for human consumption was prepared under regulatory guidelines and connected to a custom-built, MR-compatible perfusion platform supporting left ventricular function in both Langendorff and working modes. Autologous heparinized blood circulated at physiological pressures and temperatures. MR-guided catheter navigation and RF ablation were performed on a Philips 3T scanner using active catheter tracking. Native T1 and T2 mapping were acquired before and after ablation. Lesions were confirmed by histologic analysis.

**Results:**

RF ablation (50 W, 60 seconds) was successfully performed at 5 left ventricular sites. MRI showed focal reductions in T1 (936 ± 80 ms) surrounded by elevated T1 (1357 ± 18 ms) and T2 values (86 ± 10 ms) compared with nonablated myocardium (T1 1192 ± 26 ms; T2 66 ± 6 ms), consistent with necrosis and edema. Histology confirmed a necrotic core with a surrounding rim showing contraction band necrosis and erythrocyte extravasation.

**Conclusion:**

This study demonstrates the feasibility of real-time MR-guided ablation in a beating pig heart platform. The setup allows high-resolution lesion assessment and histologic correlation, supporting future developments in MR-guided therapies.

## Graphical abstract

**Figure:**
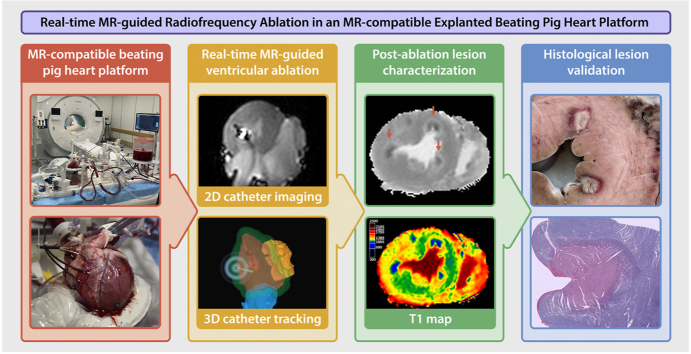

**Key Findings**

▪ This study established the feasibility of performing real-time magnetic resonance (MR)-guided radiofrequency ablation in an MR-compatible isolated beating pig heart platform.
▪ T1 and T2 mapping were able to differentiate ablation-induced changes, with histopathology confirming MR imaging’s accuracy.
▪ This platform may serve as a critical bridge between preclinical and clinical applications, allowing for the testing, validation, and optimization of MR-guided interventional techniques, with potential applications beyond ablation therapy.

## Introduction

Cardiovascular magnetic resonance imaging (MRI) has become essential for diagnosing and managing various heart conditions owing to its ability to provide detailed information on cardiovascular anatomy, function, and tissue characteristics.^1^^,^^2^ Recently, cardiovascular magnetic resonance (CMR) has emerged to support interventional procedures, leveraging its 3-dimensional (3D) visualization capabilities to create precise models, which can be integrated into the catheterization laboratory to guide interventions such as catheter ablation or endomyocardial biopsy.^3^^,^^4^

The next frontier in this evolution is interventional CMR (iCMR), where the intervention is performed under direct MRI guidance while the patient is positioned in the MRI scanner. This iCMR approach holds significant promise for a range of intricate cardiac interventions, offering simultaneous real-time 3D visualization of instruments and anatomic structures, soft-tissue characterization allowing for direct periprocedural feedback, and the advantage of eliminating exposure to ionizing radiation.^5^^,^^6^ As a result, iCMR can not only improve the outcomes of established procedures but also facilitate novel treatment options and strategies for complex arrhythmias and congenital and structural heart diseases.^7^^,^^8^

Despite the growing interest in iCMR and the technical advancements, several challenges persist. However, the validation of iCMR procedures, in particular cardiac ablation within controlled environments, along with the need for correlation of MRI readouts with histopathologic outcomes, remains elusive.^9^ The difficulty in directly comparing MRI-derived “virtual histology” with histologic samples, coupled with poorly defined quantitative MRI thresholds for distinguishing between ablated tissue and remote tissue, continues to hamper the interpretation and clinical adoption of real-time iCMR-guided cardiac ablation.^10^, ^11^, ^12^

To close the gap between magnetic resonance (MR) image features and histology, translational research is mandatory. A notable advancement in this area is the development of an MR-compatible isolated beating pig heart platform.^13^ This innovative setup simulates human physiological conditions using an explanted beating pig heart positioned inside an MRI scanner. The platform is a unique tool for testing, validating, and facilitating refinement of novel MR interventional tools and techniques, including hardware and software, devices and instruments, and advanced acquisition strategies. The platform facilitates MR-based assessments with the additional benefit of histopathologic follow-up, which is particularly interesting in the context of cardiac ablation. Moreover, the need for laboratory animals is minimized, given that the isolated heart is obtained from pigs slaughtered for human consumption, which not only reduces costs but also addresses ethical concerns associated with dedicated large animal research.^14^^,^^15^

This proof-of-concept study aimed to evaluate the feasibility of real-time MR-guided radiofrequency (RF) ablation in an MR-compatible isolated beating pig heart platform and evaluate postablation lesion characteristics within the explanted heart.

## Methods

### MR-compatible isolated beating pig heart platform

A heart was obtained from Dutch Landrace hybrid pigs slaughtered for human consumption. The procedures followed EC regulation 1069/2009 and 142/2011 concerning the use of animal materials from slaughterhouses for diagnostic and research purposes, under the oversight of the Dutch government (Ministry of Agriculture, Nature and Food Quality). These protocols were also approved by the relevant animal welfare legal authorities (Food and Consumer Product Safety Authority).

**Figure 1:**
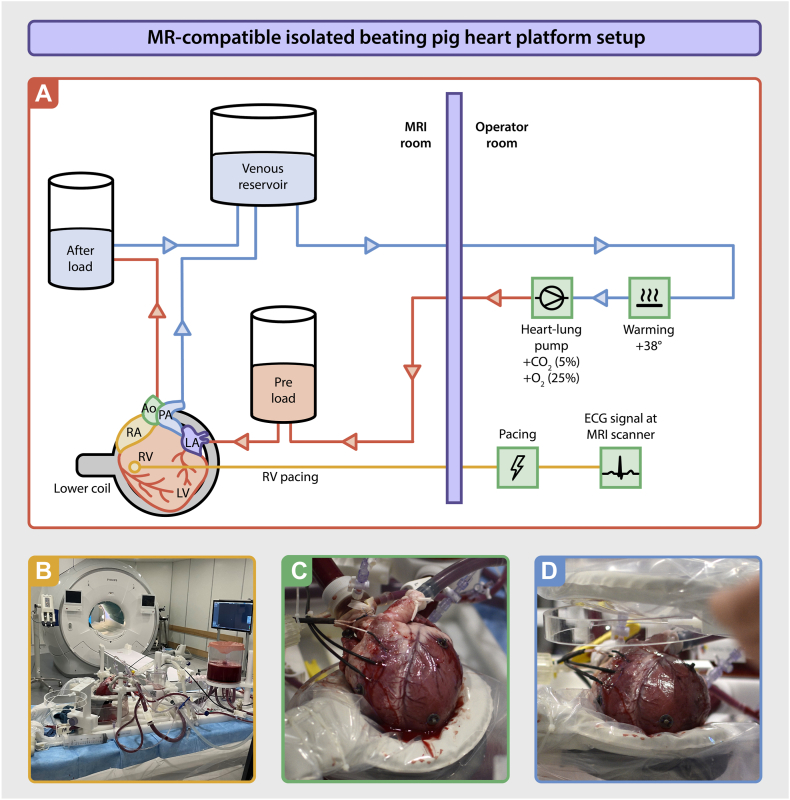
MR-compatible isolated beating pig heart platform setup. **A:** The PhysioHeart platform (LifeTec) was adapted to enable MR compatibility for detailed cardiac assessment. The explanted heart was perfused through a closed circuit, incorporating both preload and afterload to provide a physiologically realistic simulation. Venous blood (depicted in *blue*) was first heated and oxygenated outside the MR room, after which it was returned to the setup for perfusion (depicted in *red*). **B:** Photo of the MR-compatible isolated beating pig heart platform on the scanner table in the MR room. **C:** Photo of the isolated beating pig heart, resting on the posterior RF receive flex coil. **D:** Photo of the anterior RF receive flex coil placed on top of the isolated beating pig heart. Ao = aorta; CO_2_ = carbon dioxide; LA = left atrium; LV = left ventricle; PA = pulmonary artery; MR = magnetic resonance; MRI = magnetic resonance imaging; O_2_ = oxygen; PV = pulmonary vein; RF = radiofrequency; RV = right ventricle.

The animals were electrically stunned, exsanguinated, and declared dead before heart harvesting.^16^ Heparin was not administered to the animals in compliance with regulatory guidelines. Blood (5 L) was collected, and 5000 IU/L heparin was added as an anticoagulant. A parasternal incision was made, and the heart and lungs were excised en bloc by transecting the trachea, distal aorta, and the caval veins. Topical cooling was applied before aortic cannulation. 2 liters of a cold (4°C) normokalemic adenosine-lidocaine cardioplegic solution (200/500 μg/L), which contained a mixture of glucose (10 mmol/L), insulin (1 IU/L), and pyruvate (1 mmol/L) and was rendered hypocalcemic (0.6 mmol/L), was administered at a pressure of 60–80 mm Hg, as described before.^15^ The heart was placed in an iced cardioplegic solution and transported from the slaughterhouse to the hospital MRI facility. Four electrocardiography (ECG) snap buttons were sutured to the heart. Subsequently, the left atrium (LA), aorta, and pulmonary artery were cannulated and attached to the MR-compatible isolated beating pig heart platform (PhysioHeart, LifeTec Group – Resolution Medical), whereas the caval and azygos veins were sutured (Figure 1A). The PhysioHeart platform was adapted for MR compatibility using 3D printing and computer numerical control milling technology to replace all metallic mounts and connectors with plastic parts, ensuring that the heart remained in a physiological position.

The system was primed with pig heparinized diluted whole blood. The perfusate was maintained at 38°C. A gas mixture of oxygen (fraction of inspired oxygen of 20%) and carbon dioxide was used to maintain a PO_2_ of 100–200 mm Hg and PCO_2_ of 35–45 mm Hg. The biochemical composition of the perfusate was monitored using a VetScan iSTAT 1 and CG8+ cartridges (Abbott Laboratories) and maintained in a normophysiological range (pH 7.35–7.44; bicarbonate ion 20–25 mmol/L; sodium ion 135–145 mmol/L; potassium ion 3.0–5.0 mmol/L; calcium ion 1.25 mmol/L). Pyruvate (5 mmol/L) was added to the perfusate solution before reperfusion.^15^^,^^17^ Blood was warmed and oxygenated outside the MRI room, then delivered to the heart via a preload line and collected through an afterload line after circulation. To facilitate controlled rewarming and recovery, the preload and afterload lines were cross-clamped, and retrograde perfusion was performed via an aortic cannula. Hemodynamic parameters were continuously monitored and adjusted in real time using optical pressure transducers located outside the MRI room. Pacing leads were extended to enable intracardiac pacing within the MRI scanner bore (Figure 1B).

Perfusion began in Langendorff mode, targeting an aortic pressure of 70–80 mm Hg. Ventricular pacing (100 beats per minute; Medtronic) was initiated via temporary leads. A 60-minute recovery phase in Langendorff mode with controlled warming-up of the heart followed, before transitioning to working mode by filling the LA and left ventricle (LV) to initiate cardiac output. LA pressure was maintained between 10 and 20 mm Hg, and afterload was adjusted to achieve physiological cardiac output and mean aortic pressure.

### MRI scanner setup

Scanning was performed using a 3T MRI scanner (Ingenia, MR7700, Philips Healthcare), equipped with software level R12.1 and an MR therapy control interface to connect the scanner with the dedicated 3D cardiac navigation and mapping system (NorthStar, Imricor Medical Systems).

Two RF receive coils (dStream Flex M) were placed below and above the isolated beating pig heart (Figure 1C and 1D). During imaging, pacing was maintained at 100 beats per minute with MR-simulated ECG triggering synchronized to the pacing rate. Two custom-tuned 3T catheters (Vision-MR ablation catheter, Imricor) with MRI active tracking functionality, temperature sensing, and RF ablation capability were connected both to the scanner using 4 channels of a 16-channel custom-built connector box (MR Coils B.V.) and to the ablation recorder and stimulator system (Advantage-MR, Imricor Medical Systems). For RF ablation signal generation, the Advantage-MR system was connected to a dedicated RF ablation generator (HAT 500, Osypka). The 3D navigation and mapping system and the ablation recorder and stimulator system were seamlessly integrated and operated as 1 system with the MRI scanner to precisely navigate the catheters and map the RF ablation procedure.

### MRI protocol

The acquisition protocol consisted of a full-volume 3D anatomic dataset, cine imaging for functional assessment, tissue characterization, and real-time imaging for catheter navigation (sequence specifics presented in Table 1).

**Table 1:** MRI acquisition parameters for cardiac functional and tissue mapping assessment

Acquisition parameters	3D whole-heart	2D cine	T1 mapping	T2 mapping	2D real-time
Contrast	bSSFP	bSSFP	MOLLI 5 (3)3	Black blood GraSE	bSSFP
TR/TE (ms)	2.2/1.1	3.07/1.54	2.2/1.0	600/multi-TE	2.90/1.45
α (°)	70	45	20	90	45
Resolution (mm^2^)	0.97 × 0.97	0.94 × 0.94	1.17 × 1.17	1.04 × 1.04	1.56 × 1.56
Slice thickness (mm)	2	8	10	10	8
Temporal resolution	Static	31.5 ms	14 inversion delays: [0, 557] ms, ΔTI = 42.8 ms	9 echo times: [8.8 79.6] ms, ΔTE = 8.85 ms	197.5 ms
Acceleration (factor)	CS-SENSE; 2×	CS-SENSE; 2.85×	CS-SENSE; 2.4×	SENSE; 2.1×	SENSE; 2×

2D = 2-dimensional; 3D = 3-dimensional; bSSFP = balanced steady-state free precession; CS-SENSE = compressed sensing sensitivity encoding; GraSE = gradient and spin echo; MOLLI = modified look-locker inversion recovery; MRI = magnetic resonance imaging; SENSE = sensitivity encoding; TE = echo time; TR = repetition time.

#### 3D model generation

A full-volume anatomic dataset was obtained using an ECG-triggered 3D balanced steady-state free precession sequence, enabling high-resolution depiction of cardiac structures. This dataset served as input for 3D whole-heart segmentation performed with ADAS 3D software (ADAS 3D Medical). The segmented shell was then exported and uploaded into the NorthStar mapping system (Imricor Medical Systems), where it aligned perfectly with the spatial coordinates of the MRI scanner to allow real-time catheter navigation. The integration process closely follows the standard clinical workflow, with the key distinction that, in our case, the pig heart was beating but remained stationary in space owing to the absence of respiratory motion (ie, no rigid body displacement).

#### Functional imaging

Functional assessment of cardiac motion was performed with cine imaging using a balanced steady-state free precession sequence. A stack of 12 contiguous slices was acquired in short-axis orientation, spanning the LV from the base to the apex (slice thickness 8 mm; no interslice gap). Temporal resolution was optimized at 31.5 ms, with an in-plane resolution of 0.94 mm^2^. In addition, long-axis cine images were obtained in both 2- and 4-chamber orientations to evaluate longitudinal motion. LV volumes and function were assessed using CVI42 (version 5.17.1, Circle Cardiovascular Imaging Inc).

#### Tissue characterization

To assess myocardial tissue characteristics, T1 and T2 mapping were performed at baseline, immediately after ablation (acute phase), and approximately 40 minutes after ablation (subacute phase). For each time point, T1 and T2 maps were acquired in 3 contiguous midventricular short-axis slices covering a 30-mm region (slice thickness 10 mm; no gap) and a 4-chamber orientation. These images were used to assess changes in tissue characteristics induced by the ablation using CVI42. Differences between mapping values of ablation core and periphery and ablation core and reference area were assessed using the Wilcoxon signed-rank test.

#### Catheter navigation

**Figure 2:**
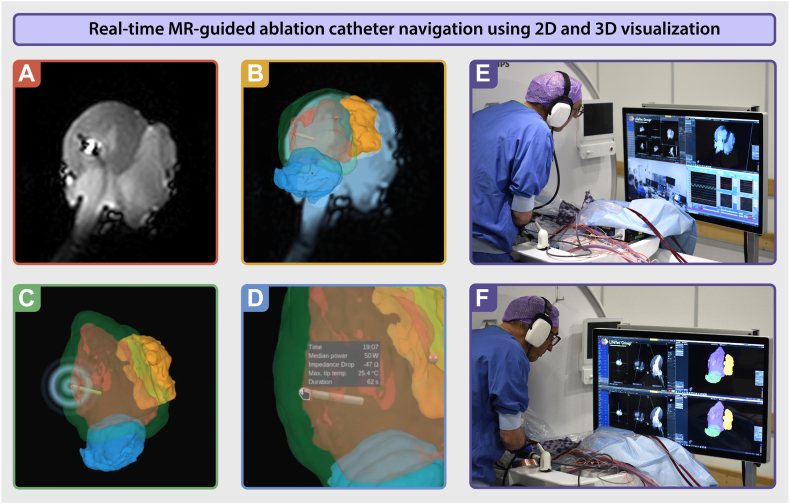
Real-time MR-guided ablation catheter navigation using 2D and 3D visualization. **A:** Real-time 2D active catheter imaging within the pig heart. The ablation catheter stands out owing to a clear susceptibility artifact, with microcoils near the catheter tip producing a pronounced “halo” effect. **B:** A combination of 2D active catheter imaging and 3D active catheter tracking displayed as an overlay within the NorthStar system. The rendered tip of the catheter displayed can be seen in the left ventricular cavity. **C:** Ablation targeting the lateral wall of the left ventricle. The catheter projection is visible within the 3D anatomic shell, showing precise positioning. **D:** Ablation details become visible when the mouse hovers over the ablation point, providing contextual information. **E** and **F:** Images of the interventionalist navigating the ablation catheter inside the left ventricle, guided by real-time MR imaging and the 3D anatomic shell. MR = magnetic resonance; 2D = 2-dimensional; 3D = 3-dimensional.

The MR-compatible irrigated ablation catheter was introduced into the LA through an MR-compatible sheath via the right pulmonary vein. The catheter tip, with integrated MR RF receiver coils, appeared bright on MR images, allowing for real-time visualization during navigation (ie, active catheter imaging). Initially, active 2-dimensional catheter imaging was used to guide the catheter from the LA into the LV. After entry into the LV, a combination of active 2-dimensional catheter imaging and 3D tracking in the previously created 3D anatomic model enabled precise guidance during ablation procedures (Figure 2).

#### Ablation protocol

After obtaining baseline imaging, the perfusion mode was reverted to Langendorff to optimize pig heart stability during the ablation procedure and reduce susceptibility to ablation-induced arrhythmias. Thereafter, the RF ablation was performed with a power setting of 50 W applied for a duration of 60 seconds per target. Five distinct ablation targets were selected within the LV myocardium, encompassing the ventricular septum and lateral wall, with all sites located within regions characterized by baseline T1 and T2 mapping.

### Histologic analysis

Immediately after imaging, the heart was fixed in buffered formaldehyde. Transverse cross-sections of the myocardium were then obtained for gross pathologic examination. 2 lesions were isolated from these sections and further divided into 2-mm slices. Histologic slides from these regions were stained with phosphotungstic acid-hematoxylin (PTAH) for detailed microscopic evaluation. The stained slides were digitized using a high-resolution whole-slide imaging system (IntelliSite Ultra Fast Scanner, Philips Healthcare) for subsequent visual analysis.

## Results

### Preablation imaging

Functional imaging before ablation revealed abnormal LV wall motion characterized by late contraction of the lateral wall (Supplemental Video 1). LV end systolic volume, LV end diastolic volume, and LV ejection fraction were 78 mL, 102 mL, and 24%, respectively. Native myocardial T1 was 1360 ± 92 ms, and T2 was 55 ± 15 ms.

### Ventricular ablation

Once the ablation catheter was positioned, the initial ablation point in the septum was applied and ventricular fibrillation immediately occurred. No further action was taken to restore rhythm. Subsequently, 4 additional ablation points were delivered at various locations in the LV, each using the same power setting. The total procedural time was 15 minutes. Acute postablation imaging was performed directly after the last ablation point.

### Acute postablation imaging

**Figure 3:**
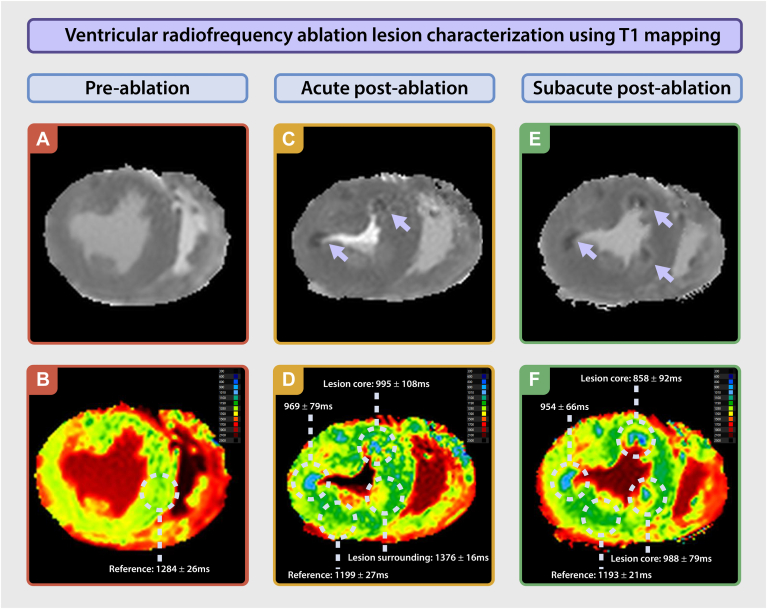
Ventricular radiofrequency ablation lesion characterization using T1 mapping. **A, C,** and **E:** Ventricular short-axis *gray*-scale T1 mapping acquisition data, showing the preablation baseline (**A**) and postablation images (**C** and **E**). *Arrows* highlight ablation lesions, which appear as a low signal-intensity core surrounded by a high signal-intensity border. **B, D,** and **F:** Ventricular short-axis parametric T1 maps, illustrating the preablation baseline (**B**) and postablation images (**D** and **F**). Regions of interest indicate T1 mapping values of the lesion cores used in quantification.

During ventricular fibrillation, postablation imaging demonstrated an LV cavity volume of 52 mL. Directly after ablation, reference (nonablated) T1 was 1192 ± 26 ms, and T2 was 66 ± 6 ms. The ablated regions exhibited a core region with a shorter T1 of 936 ± 80 ms (core vs reference; *P* < .01), surrounded by a smaller region with a longer T1 of 1357 ± 18 ms (core vs periphery; *P* < .01) (Figure 3). T2 mapping revealed a central ablation core with T2 of 65 ± 8 ms, which was similar to the reference myocardium (66 ± 6 ms; *P* = .83), whereas the surrounding rim demonstrated higher T2 values (86 ± 10 ms; *P* < .01), suggestive of edema.

### Subacute postablation imaging

After completing acute postablation imaging, the MR-compatible pig heart platform was removed from the scanner for successful defibrillation, which was performed 1 time using 30 J. The platform was subsequently repositioned into the scanner, and a second series of postablation imaging was conducted 40 minutes after the ablation procedure, while the heart was beating. Subacute imaging revealed an LV cavity volume of 80 mL, along with marked late contraction of the LV wall, impairing effective ejection of blood (LV ejection fraction 8%). Reference (nonablated) T1 was 1193 ± 21 ms, and T2 was 63 ± 9 ms. The ablated regions continued to demonstrate a core with a short T1 of 954 ± 89 ms, surrounded by areas with a long T1 of 1360 ± 68 ms (Figure 3). T2 mapping showed a core with a slightly longer T2 of 71 ± 8 ms than the reference region of interest, with adjacent areas exhibiting an elevated T2 of approximately 82 ± 8 ms.

### Macroscopic and microscopic analysis

**Figure 4:**
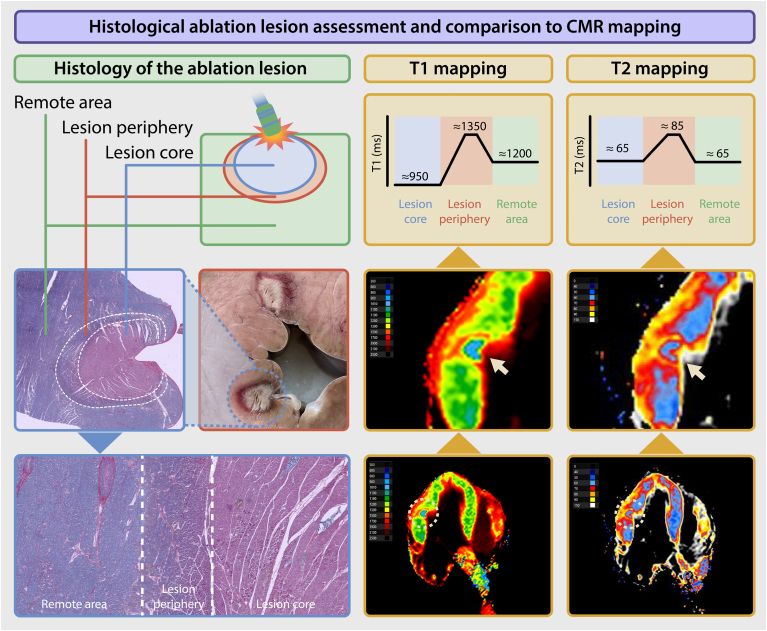
Histologic ablation lesion assessment and comparison with CMR mapping. A histologic representation of the ablation lesion, highlighting a distinct biphasic pattern comprising a lesion core and peripheral zone, as demonstrated by gross pathology and phosphotungstic acid-hematoxylin staining. This biphasic architecture is corroborated by corresponding T1 and T2 parametric maps, underscoring the integration of imaging and histologic findings. CMR = cardiovascular magnetic resonance.

Macroscopic evaluation of all 5 RF catheter ablation lesions demonstrated a well-demarcated gray core, encircled by a darker red rim (Figure 4). Microscopically, the central zone consisted of necrotic cardiomyocytes and extravasation of erythrocytes (pink staining in the PTAH staining). This necrotic area was surrounded by a border zone of cardiomyocytes with contraction band necrosis and extravasation of erythrocytes (purple in the PTAH staining).

## Discussion

This study establishes the feasibility of real-time MR-guided RF ablation within an MR-compatible isolated beating pig heart platform. This innovative experimental platform integrates advanced imaging and catheter navigation techniques with histologic validation, enabling lesion visualization and comprehensive characterization that are difficult to achieve in complex in vivo settings. Serving as a critical translational bridge between preclinical research and clinical application, this setup enables testing, validation, and optimization of MR-guided interventional techniques. Beyond myocardial ablation guidance, the platform has potential applications in developing and integrating other MR-compatible devices, such as bioptomes and ECG devices, further advancing its translational and clinical significance. Recent clinical studies have demonstrated the feasibility of MR-guided ablation in humans.^18^, ^19^, ^20^, ^21^ Our study complements these efforts by providing a reproducible preclinical platform in which novel imaging techniques, MR-compatible tools, and lesion creation strategies can be tested before in vivo or clinical translation.

CMR imaging has emerged as a powerful tool for lesion visualization during ablation.^9^^,^^22^ Characterization of ablation lesions using CMR provides invaluable insights, which can be subsequently validated through a histopathologic analysis of the pig heart. This integration is critical for assessing MRI-based lesion assessments and introducing quantitative markers that accurately distinguish ablated from nonablated myocardial tissue. Furthermore, the platform facilitates the evaluation of various ablation parameters, including higher power settings, extended ablation duration, and the modification of scar tissue. These capabilities address the ongoing challenge of correlating MRI-derived “virtual histology” with definitive histopathologic outcomes, as well as the absence of standardized MRI criteria for evaluating ablation efficacy. As such, the MR-compatible explanted beating pig heart platform holds promise in refining and standardizing MR-guided interventions, with the potential to significantly improve patient outcomes.

Preablation mapping analysis of the explanted beating pig heart demonstrated globally elevated T1 and T2 values compared with reference values at 3T for human hearts, which are similar for porcine hearts.^23^^,^^24^ This increase may be attributable to the catecholamine release owing to stress induced by the slaughter process, the cooling and rewarming processes of the explanted heart, ischemic stunning, and possibly in vivo immune and inflammatory responses induced before slaughter. To assess the ablation effects, we compared postablation imaging findings from ablated myocardial areas with a reference nonablated myocardium. Postablation imaging revealed that T1 mapping of the nonablated myocardium showed a reduction in T1 values after ablation. This reduction could reflect diminished perfusion, potentially arising owing to ventricular fibrillation, microvascular compromise, or reduced metabolic activity.^25^^,^^26^ In contrast, T2 mapping demonstrated elevated values in the nonablated myocardium, suggestive of edema formation. These increased T2 values likely indicate an intricate interplay of ischemic injury, inflammation, and tissue degeneration within the nonablated myocardium.^27^^,^^28^

The ablation lesion imaging findings support the utility of MRI for characterizing acute tissue changes after ablation. The observed acute changes in T1 and T2 values in the ablation zones align with previous reports on the histopathologic response to RF energy.^29^ These findings also align with previous experimental work demonstrating MR visualization of evolving RF lesions in vivo.^30^ The core of the ablation lesion demonstrated a short T1 value relative to a reference nonablated myocardium. This may be attributable to tissue necrosis and early fibrosis, processes that result in the accumulation of macromolecules, protein cross-linking, and cellular debris, thereby shortening the T1. In addition, hemorrhagic components within the core, resulting from ablation-induced vascular damage, can potentially further reduce T1 values owing to the paramagnetic effects of blood breakdown products, such as deoxyhemoglobin and methemoglobin.^31^^,^^32^ In contrast, the surrounding periphery of the lesion is characterized by a long T1 value. This observation is likely caused by the presence of inflammation and interstitial edema in the zone surrounding the necrotic core, where extracellular water accumulation leads to an increase in T1 values.

Regarding T2 parametric mapping, the core of the lesion showed values that are similar to those of a reference nonablated myocardium, despite the presence of edema and necrosis. This may be explained by the balancing effects of coexisting edema, which increases T2, and hemorrhage, which decreases T2 through paramagnetic effects, effectively cancelling each other out. However, in the periphery of the lesion, there was typically an elevated T2, likely attributable to interstitial edema, which is a common inflammatory response after myocardial injury. This peripheral edema increases the water content in the tissue, prolonging the T2 values compared with reference myocardium.

Subsequent histologic analysis also demonstrated a biphasic pattern, with macroscopic evaluation of the RF catheter ablation lesions revealing a well-demarcated gray necrotic core surrounded by a rim. These regions corresponded to distinct histopathologic features: the central core exhibited necrosis and was surrounded by cardiomyocytes with contraction band necrosis with extravasation of erythrocytes. This aligns with the observed low T1 values in the lesion core, which are likely driven by the accumulation of macromolecules and paramagnetic effects from hemorrhagic byproducts such as deoxyhemoglobin and methemoglobin. These findings underscore the utility of MRI for distinguishing the acute pathophysiological responses to RF ablation at a tissue level. Although lesion depth and dimensions could theoretically be quantified, such measurements could be imprecise in this exploratory setup. The spatial coordinates of histologic sections and MR images may not perfectly overlap, and the relatively thick short-axis slices acquired for the T1 maps inherently average the signal over the slice. Therefore, we restricted our analysis to a qualitative comparison between imaging and histology in this exploratory setup.

### Limitations

Despite its promise, there are still aspects of this isolated beating heart platform that could be improved. The platform does not reproduce the systemic inflammatory response, respiratory motion, or in vivo impedance conditions, which may affect ablation lesion formation and translation to the clinical situation. Consequently, lesion size and tissue response may differ from in vivo models. Therefore, the platform may primarily serve as a translational testbed for visualization, catheter navigation, sequence development, and testing new ablation strategies rather than for exact lesion characterization. Once optimized, these developments can be transferred to in vivo large animal studies and eventually to human applications. Besides, our experiment was performed in a normal pig heart without an arrhythmogenic substrate. Lesion formation and tissue characterization within chronic infarct or fibrotic regions may differ substantially and represent an important area for future research. Moreover, a key challenge lies in the electromechanical instability owing to the absence of the complex hormonal and biochemical milieu inherent to a living organism. This limitation may affect reproducibility, particularly during prolonged or repetitive procedures. Nevertheless, in our study, we successfully conducted experiments lasting more than an hour. After baseline imaging, we transitioned to Langendorff perfusion mode to enhance stability of the pig heart during ablation, given concerns about ablation-induced arrhythmias. Despite this precaution, ventricular fibrillation occurred after the initial ablation, and defibrillation could only be applied after removing the platform from the scanner. Future advancements, including optimized electromechanical stability and in-bore defibrillation protocols, could extend the functional window of this platform in working mode. Notably, recent work has demonstrated the stability of this platform for up to 9 hours, including 8 hours in working mode, underscoring the robustness of this platform.^15^ Importantly, the heart used in our experiment was not treated with antiarrhythmic agents, such as lidocaine or amiodarone, which might have further enhanced electrophysiological stability. Furthermore, although our ablations were performed in the LV, transitioning to atrial ablation represents a key focus for future research. Moreover, although testing additional MRI sequences such as non-contrast-agent-enhanced T1-weighted imaging using long inversion time, postcontrast T1-weighted short inversion time, late gadolinium enhancement imaging, or thermographic imaging would have been valuable, this was not feasible within the scope of our study, given that it was part of a larger consortium project in which time and scanner availability had to be shared with other experimental protocols.^11^^,^^32^, ^33^, ^34^ However, detailed visualization of ablation lesions in the thin-walled atrial wall will bring new challenges. Finally, although the system enables precise, real-time visualization of cardiac interventions, its reliance on specialized equipment, multidisciplinary expertise, and substantial resources may pose barriers to widespread adoption.

## Conclusion

The development of this MR-compatible working heart platform represents a major advance in MR-guided cardiac interventions. This platform facilitates real-time catheter tracking, enables precise lesion characterization, and provides a controlled environment for the evaluation of novel techniques, thereby laying a foundation for future innovations in MR-guided cardiac interventions. The imaging characteristics observed in response to RF ablation during the experiment are consistent with findings from human studies, underscoring its translational relevance. Nonetheless, several challenges remain, including electrophysiological instability and the lack of autonomic innervation. Future research should focus on overcoming these limitations and expanding the clinical applicability of MR-guided cardiac procedures, with the ultimate goal of enhancing the reliability and translational utility of this platform.

## Disclosures

Bart L.M. Smeets and Renate W. Boekhoven are employees of LifeTec Group B.V., Eindhoven, The Netherlands. Daniel Sunnarborg is an employee of Imricor Medical Systems, Burnsville, MN. Jouke Smink is an employee of Philips Healthcare, Best, The Netherlands. All other authors have no conflicts of interest to disclose.
