# Supplementary material for: Real-time magnetic resonance-guided radiofrequency ablation and lesion evaluation in an magnetic resonance-compatible isolated beating pig heart platform
Source: Heart Rhythm O2. 2025 Sep 19;6(12):2001–10. doi: 10.1016/j.hroo.2025.09.014 (PMC12800846; doi:10.1016/j.hroo.2025.09.014)
Supplement: Supplementary material [file mmc1.docx]

**Supplementary material file**

Screen recording of long-axis and short-axis cine imaging before ablation in the MR-compatible explanted beating pig heart.
